# A new mode of contrast in biological second harmonic generation microscopy

**DOI:** 10.1038/s41598-017-13752-y

**Published:** 2017-10-17

**Authors:** Nicola H. Green, Robin M. Delaine-Smith, Hannah J. Askew, Robert Byers, Gwendolen C. Reilly, Stephen J. Matcher

**Affiliations:** 1 Department of Materials Science and Engineering, Kroto Research Institute, North Campus, Broad Lane, University of Sheffield, Sheffield, S3 7HQ UK; 2 The School of Engineering and Materials Science, Queen Mary, University of London, Mile End Road, London, E1 4NS UK; 3 The University of Sheffield Advanced Manufacturing Research Centre (AMRC) with Boeing Factory 2050, Sheffield Business Park, Europa Avenue, Sheffield, S9 1ZA UK; 4 Department of Electronic and Electrical Engineering, Kroto Research Institute, North Campus, Broad Lane, University of Sheffield, Sheffield, S3 7HQ UK; 5 INSIGNEO Institute for in silico Medicine, The Pam Liversidge Building, Sir Robert Hadfield Building, Mappin Street, Sheffield, S1 3JD UK

## Abstract

Enhanced image contrast in biological second harmonic imaging microscopy (SHIM) has previously been reported via quantitative assessments of forward- to epi-generated signal intensity ratio and by polarization analysis. Here we demonstrate a new form of contrast: the material-specific, wavelength-dependence of epi-generated second harmonic generation (SHG) excitation efficiency, and discriminate collagen and myosin by ratiometric epi-generated SHG images at 920 nm and 860 nm. Collagen shows increased SHG intensity at 920 nm, while little difference is detected between the two for myosin; allowing SHIM to characterize different SHG-generating components within a complex biological sample. We propose that momentum-space mapping of the second-order non-linear structure factor is the source of this contrast and develop a model for the forward and epi-generated SHG wavelength-dependence. Our model demonstrates that even very small changes in the assumed material fibrillar structure can produce large changes in the wavelength-dependency of epi-generated SHG. However, in the case of forward SHG, although the same changes impact upon absolute intensity at a given wavelength, they have very little effect on wavelength-dependency beyond the expected monotonic fall. We also propose that this difference between forward and epi-generated SHG provides an explanation for many of the wavelength-dependency discrepancies in the published literature.

## Introduction

Second harmonic imaging microscopy (SHIM) involves illuminating non-centrosymmetric structures with an ultrashort pulse of focused laser radiation: the resulting transient power densities can then become so high (10^18^ W m^−2^) that second-order non-linear frequency doubling occurs. Second harmonic generation (SHG) is mediated by the non-linear second-order susceptibility tensor χ^(2)^, which is unusually high in collagen and myosin^1^. Using near-infrared excitation wavelengths, SHIM penetrates deep into tissues and constructs three-dimensional images of specimens, making it ideal for non-destructive tissue mapping. SHG intensity is influenced by the type^2^, quantity and organization^3^ of the collagen present. SHG polarimetry can determine the collagen fibre orientation^4,5^ and potentially discriminate myosin, type-I and type-II collagen^2,6^. Another potential contrast mechanism is the ratio of forward- to epi-generated signal^5,7^. However, wavelength-dependent SHG excitation efficiency has been little studied, with conflicting results and it has not been clear to date whether this signal carries any spectroscopic information.

The full permutation symmetry (Kleinman symmetry) is widely assumed in biological SHIM and requires that χ^(2)^ is independent of wavelength^8^. However, even in this case, wavelength-dependence will arise from factors including the variation in dipole oscillator radiated field amplitude with frequency and 1/λ^2^ fall in focal spot intensity due to diffraction. Consequently, this assumption predicts SHG efficiency falls monotonically with wavelength in a material-independent way. Shen *et al*.^9^ produced results that support this assumption when measuring 10 µm sections of mouse tail tendon, in both the forward- and epi- directions. Cox *et al*.^10^ have stated that there is no wavelength dependency when imaging thin sections of collagen tissue; Zipfel *et al*.^11^ measured SHG emission from collagen I gels and rat tail tendon sections using excitation wavelengths between 730 nm and 980 nm, describing an identical monotonic fall with wavelength for both materials, although this work appears to assess wavelength dependency for forward generated SHG only. However, some groups report more complex behaviours. For example, Zoumi *et al*.^12^ investigated epi-detected SHG efficiency from a synthetic skin model excited between 730 and 880 nm, reporting signals that peaked at 800 nm before plateauing at 760 nm. Theodossiou *et al*.^13^, meanwhile, observed that forward-detected SHG from rat tendon was very low at 800 nm reaching a maximum at 880 nm, while epi-detected SHG displayed multiple peaks of comparable amplitude. Deng *et al*.^14^ report epi-generated material dependent wavelength dependency, with an increase in intensity with wavelength for stromal tissue and an 850 nm maximum for hyperplastic parenchyma. Using a Zeiss LSM510 META system, we previously made epi-direction measurements on dermis, intervertebral disk, bovine tendon and various electrospun polymer scaffolds used in tissue-engineering and also found more complex behavior^15^. Notably, we reported SHG efficiency from type I collagen-containing samples appeared to increase at longer wavelengths, directly contradicting expectations based on the Kleinman symmetry.

### Theory

Freund *et al*.^16^ expressed the far-field SHG intensity *S*_2_ under plane wave illumination as: -

1 $${S}_{2}({\rm{\Delta }}k)\propto {|G({\rm{\Delta }}k)|}^{2}$$

2 $$G({\rm{\Delta }}k)={\iiint }_{vol}\beta (r)\exp (-i{\rm{\Delta }}k\cdot r){d}^{3}r$$

where ${\rm{\Delta }}k=2{k}_{1}-{k}_{2}$ is the momentum shift. *G* is the second-order non-linear structure factor and is the 3-D Fourier Transform of the hyperpolarizability distribution β(**r**). In the forward-direction $|{\rm{\Delta }}k|=4\pi (n(\lambda /2)-n(\lambda ))/\lambda$, whilst in the epi-direction $|{\rm{\Delta }}k|=4\pi (n(\lambda /2)+n(\lambda ))/\lambda$. Since $|{\rm{\Delta }}k|$ is wavelength-dependent, varying the excitation wavelength causes a translation through momentum (${\rm{\Delta }}{\bf{k}}$) space. The overall SHG intensity can therefore change if *G* changes, even if χ^(2)^ remains constant.

Literature data for *n*(λ) for water^17^ demonstrates the excitation wavelength range 0.76–1.0 µm corresponds to a momentum-space range of only 0.25 µm^−1^ in the forward-direction but 12 µm^−1^ in the epi-direction. Features in *G* due to the microstructure of β(**r**) are therefore much more likely to be resolved by a wavelength scan of epi-generated rather than forward-generated SHG.

It is important to include spatial field confinement due to tight focusing by an objective of numerical aperture NA, because this effectively apodizes β(**r**) and thus smears out features in *G*. The model used is based on Boyd’s paraxial solution to Gaussian beam propagation through a non-linear medium^18^. Whilst more accurate models have been recently developed^1,19^, Boyd’s approach results in a Fourier-transform relation between the axial distribution χ^(2)^(*z*) and the 1-D structure factor (Equation 3) which is intuitive and, we believe, captures the essence of our argument: -

3 $$G({\rm{\Delta }}k)={\int }_{-\infty }^{+\infty }\frac{{\chi }^{(2)}(z)\exp (i{\rm{\Delta }}kz)}{1+i\zeta }dz$$

where $$\zeta =\pi N{A}^{2}\cdot z/(n\lambda )$$.

## Results

### Investigation of wavelength dependency of collagen using spectral stacks

**Figure 1:**
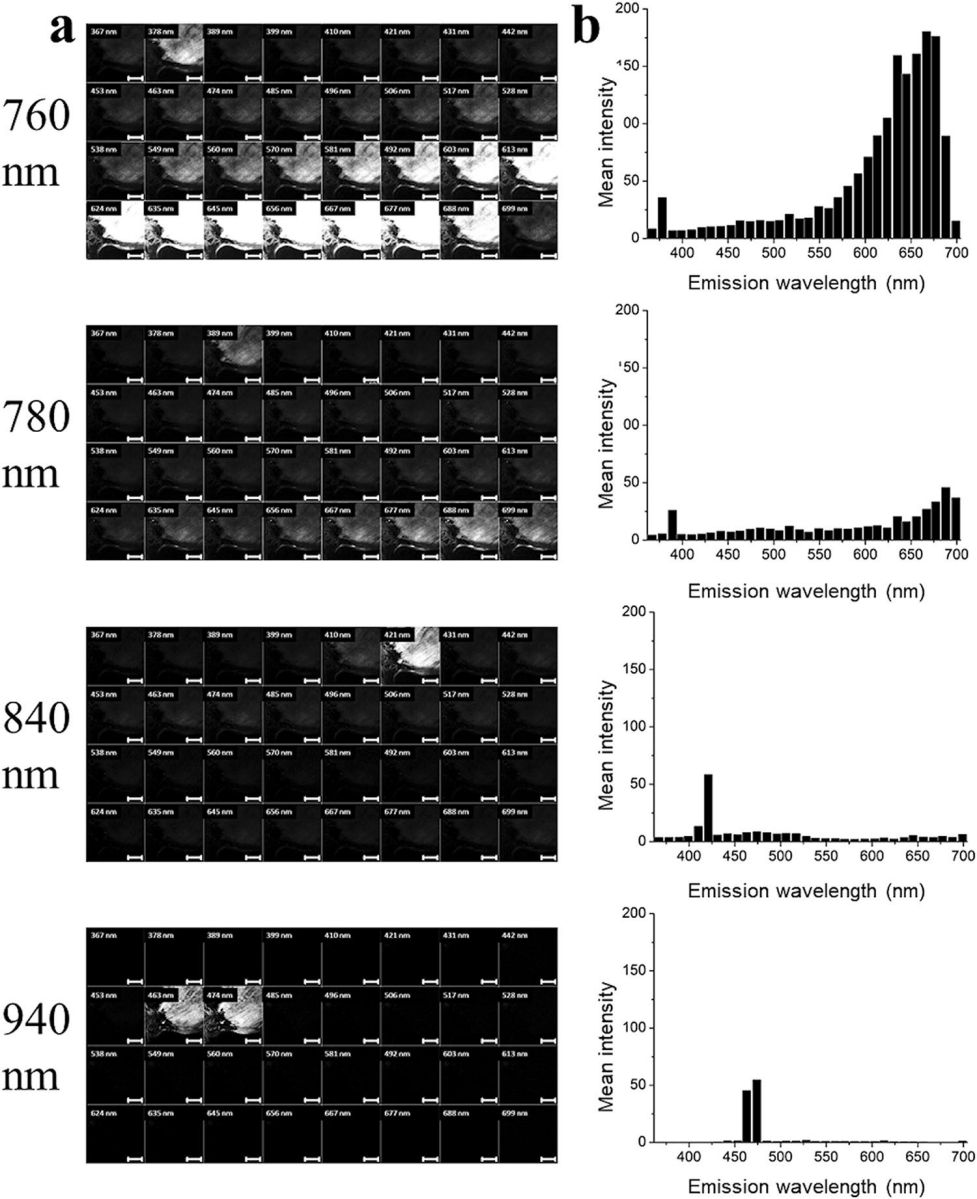
Spectral stacks obtained from collagen. Examples of spectral stack images of a single plane from bovine tendon (**a**) using 760 nm, 780 nm, 840 nm and 940 nm illumination wavelengths. Each of the 32 sub-panels shows the image collected in consecutive 10.8 nm pass-bands between 367 and 699 nm. Scale bar indicates 50 µm. Mean emission spectra (n = 3) are derived from these images (**b**).

Complete spectral stacks of bovine tendon were first analysed to provide information on the emission profile at each wavelength and confirm that the signal observed was SHG. Average illumination power onto the sample was carefully maintained at a constant value as excitation wavelength varied, by calibrating using a thermal power meter at the objective focus. Figure 1a shows example images from selected wavelengths (760, 780, 840 and 940 nm). Each 4 row × 8 column montage is a multispectral data stack with 10.8 nm spectral resolution. Also shown are the emission profiles at these wavelengths (Fig. 1b). Illumination at 760 nm resulted in the two-photon excitation fluorescence (TPEF) signal dominating the response, shown by the signal detected at longer wavelengths than the SHG signal. However, a definite SHG signal was still apparent in the 378/10.8 nm image. Illumination at 780 nm resulted in the continued presence of an SHG signal (389/10.8 nm) together with an obvious reduction in TPEF. This TPEF totally disappeared at longer illumination wavelengths, although an SHG signal is still observed with 840 nm (421/10.8 nm) and 940 nm (453/10.8 nm and 474/10.8 nm) illumination.

**Figure 2:**
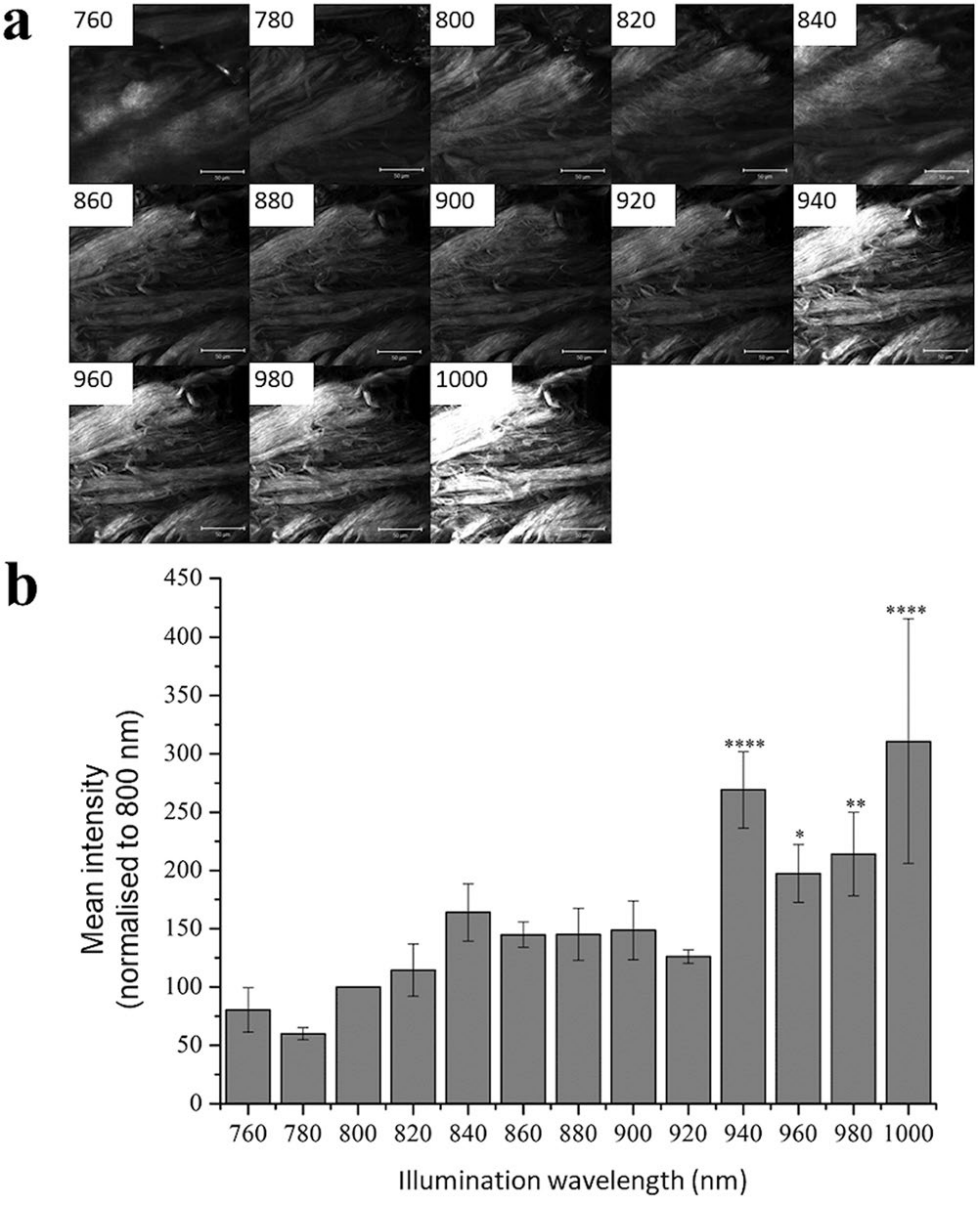
Collagen SHG intensity between 760 and 1000 nm excitation. SHG images of a single plane within bovine tendon (**a**) for excitation wavelengths between 760 and 1000 nm with 20 nm increment. Scale bar 50 µm. SHG signal intensity normalized against the intensity for the same sample at 800 nm, determined for the whole field of view (n = 3) at illumination wavelengths between 760 and 1000 nm (**b**). Values shown are mean+/− S.E.M, n = 3. Results are analysed by ANOVA and Bonferroni post-hoc comparison, with significance differences to 800 nm result indicated by ****P ≤ 0.001, **P ≤ 0.01, *P ≤ 0.05. Scale bar indicates 50 µm.

Three fields of view were then imaged at each wavelength (Fig. 2a) and the mean SHG signal intensity for each field of view determined using ImageJ^20^ (Fig. 2b). To account for variations in signal intensity between different fields of view within the same sample type, the SHG intensities were normalised as a percentage of the intensity determined at 800 nm. This wavelength was chosen since it is a popular choice for collagen SHG imaging^12,21^.

The epi-generated SHG signal intensity oscillated over the illumination wavelength range tested. Illumination wavelengths of >940 nm produced SHG signals significantly greater than that observed at 800 nm; with peaks at 940 and 1000 nm illumination. The strongest signal was obtained at 1000 nm, however there was also the most inter-sample variability at this wavelength^15^.

### Investigation of wavelength dependency using fixed filters

**Figure 3:**
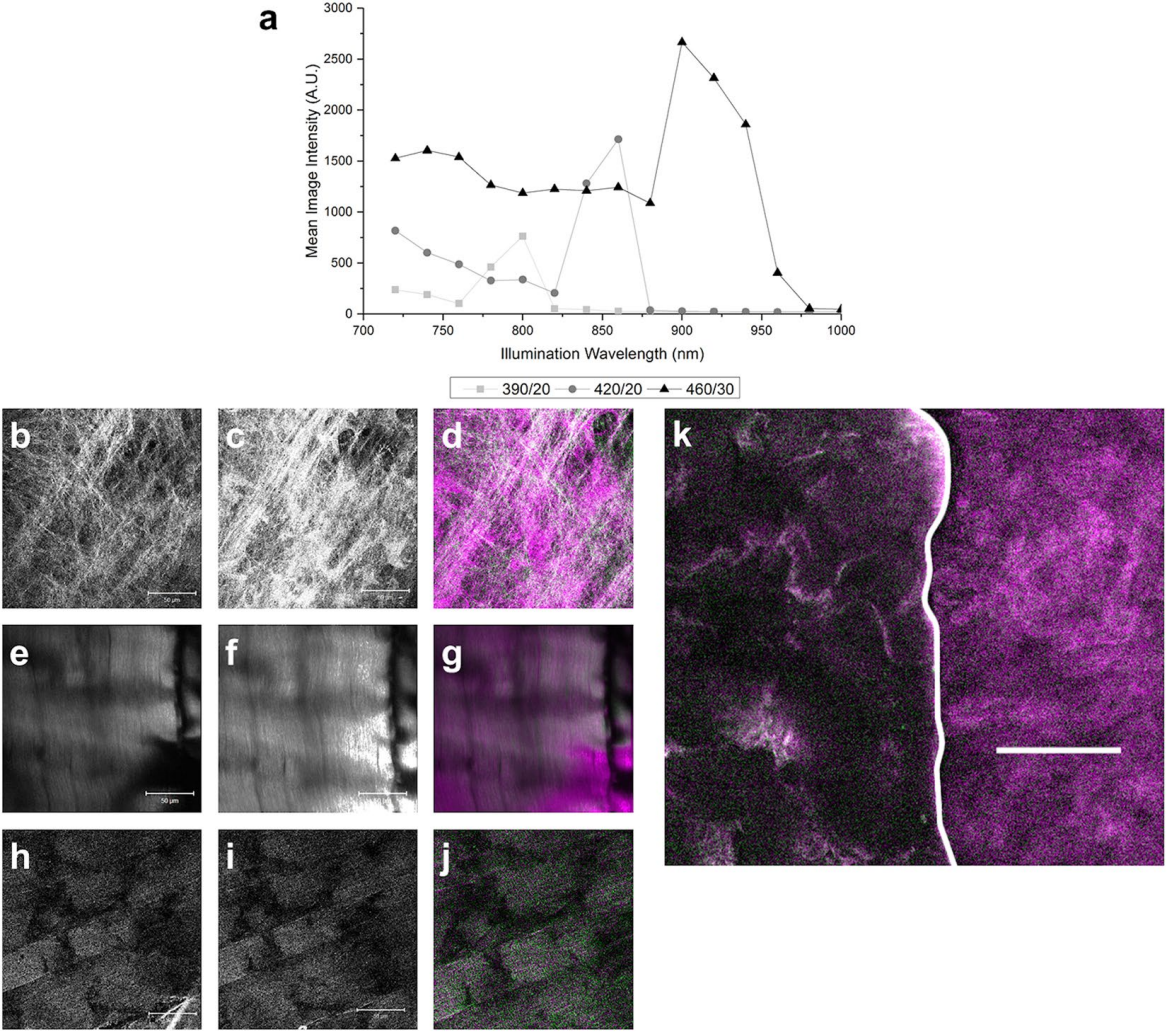
Comparison between collagen and myosin SHG intensity response to excitation wavelength. Images obtained in the epi-direction from type I collagen samples using 720 to 1000 nm wavelengths. Narrow band detection filters were employed (390/20, 420/20 and 460/30 nm) at each wavelength and the image intensity determined (**a**). Representative images of collagen generated by fibroblasts (**b**,**c**), or bovine tendon (**e**,**f**) and myosin (**h**,**i**) at λex 860 nm, λem 410–430 nm (**b**,**e**,**h**) and λex 920 nm, λem 445–475 nm (**c**,**f**,**i**). False-coloured ratio images were generated for collagen from fibroblasts (**d**), or in bovine tendon (**g**) or for myosin (**j**) showing pixels with greater intensity at 920 nm than 860 nm as purple, the converse as green, and equal intensity at both wavelengths as grayscale. A false-coloured ratio image of a combined sample of collagen and myosin (**k**), with collagen on the right and myosin on the left of the sample shows a similar difference in the ratio of the two signals. Scale bar 50 µm.

To demonstrate that these and the previously reported increases in SHG intensity^15^ were not artefacts from the manually defined filters in the Zeiss META detector, and to show that the method can be applied to microscopes without this type of detector, the experiment was repeated using customized narrow bandwidth filters (380–400 nm, 410–430 nm or 445–475 nm) inserted into the light path of a standard photomultiplier tube detection channel of our LSM510. The intensity of the image of fibroblast-generated type I collagen was determined with each filter using excitation wavelengths between 740 and 1000 nm (Fig. 3a). The integrated intensity of the image shows a peak when the excitation wavelength equals twice the filter centre-wavelength, again implying SHG emission. Emission for shorter excitation wavelengths is likely due to collagen TPEF and, as expected, the intensity for this rapidly falls to zero for longer wavelengths. To ensure maximum rejection of TPEF, we measured SHG when the excitation wavelength was exactly twice the filter centre wavelength. Similar to results derived using the META detector (ref.^15^ and Fig. 2), collagen SHG intensity was seen to increase with longer excitation wavelengths. This was the case for both collagen generated by cultured fibroblasts (Fig. 3b–d) and bovine tendon (Fig. 3e–g). Images obtained at 860 nm (Fig. 3b,e) and 920 nm (Fig. 3c,f) illumination were software-registered and used to generate a ratiometric overlay (Fig. 3d,g). The false-colour intensity images highlight the differences in pixel intensity between the 920 nm and 860 nm images. In these images purple indicates pixels of greater intensity in the 920-nm image and green indicates pixels of greater intensity at 860 nm. The collagen images clearly show many pixels are purple as a result of greater intensity in the 920-nm generated images.

Repeating this ratio analysis on SHG images of myosin demonstrates a different wavelength dependence. In this case, little difference was detected for myosin between the epi-generated SHG signal intensity using 920 nm (Fig. 3h) and 860 nm (Fig. 3i) excitation, and as result most pixels are greyscale (Fig. 3j).

A similar false-coloured image of a combined sample of collagen and myosin (Fig. 3k), with tendon on the right of the sample and muscle on the left indicates that, even when imaging the two components within the same sample, there is a difference in the ratios of the signal intensities. The collagen fibres in the tendon show a greater signal intensity at 920 nm excitation, demonstrated by the majority of purple pixels, while the increase in white and green pixels within the myosin region of the sample indicates that this is not the case for myosin. Within the muscle sample, fibres which generate a stronger SHG signal at 920 nm, and thus appear purple, can be observed and we propose that these are the collagen containing connective tissue found in muscle.

### Calibration of the wavelength-dependent response in the emission and detection paths

Since it could be argued that changes in SHG intensity at different wavelengths result from wavelength-dependent variations in the excitation efficiency (e.g. pulse stretching) or detection efficiency (e.g. incorrect software calibration of the detector DQE, etc.), the system response was compared to external calibrations in two ways.

**Figure 4:**
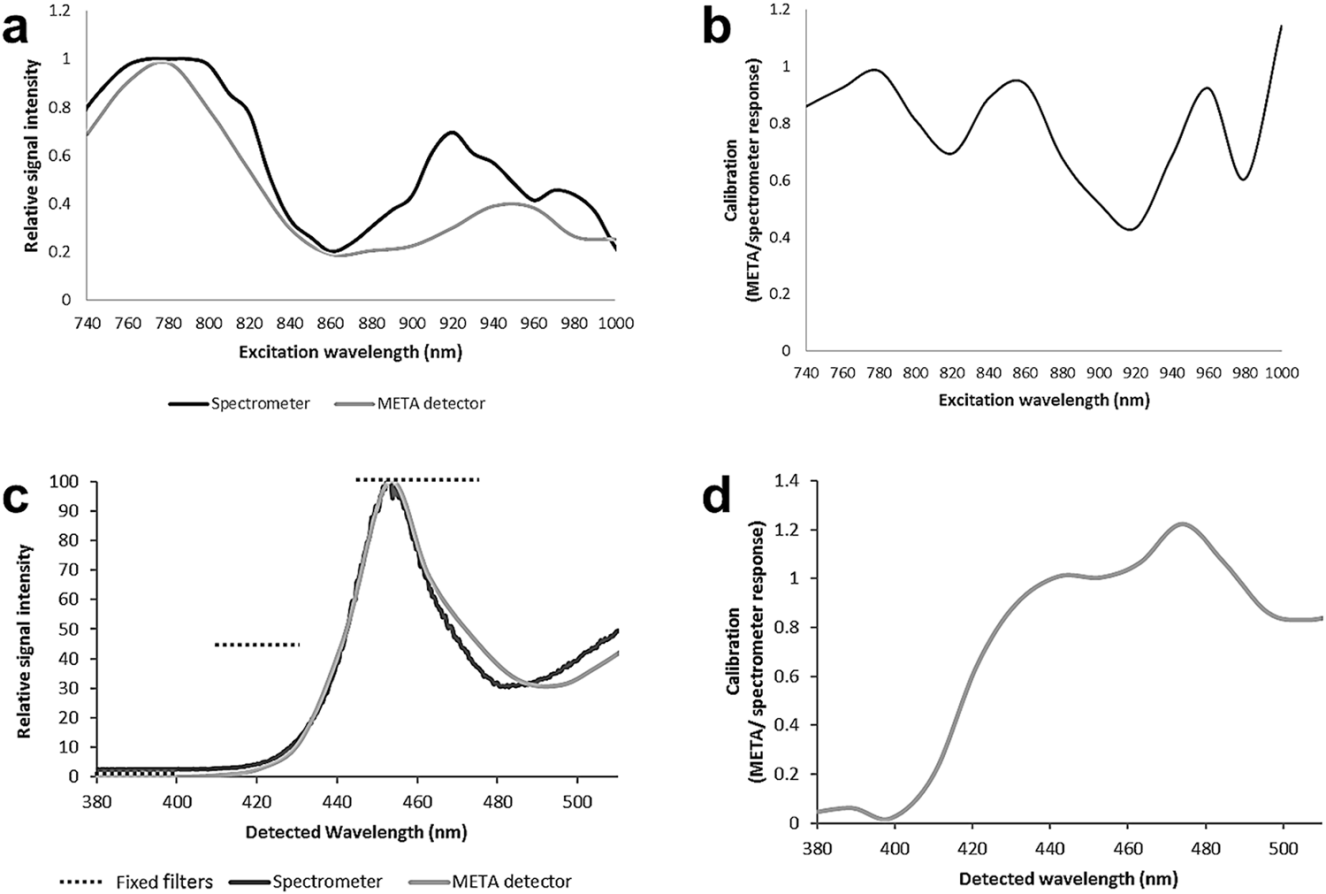
Calibration of the wavelength-dependent response in the emission and detection paths. The published two-photon excitation cross section of fluorescein compared to that obtained from the META detector (**a**) and the calibration curve derived from this data (**b**). The output spectrum (**c**) obtained from an LED torch at the wavelengths used within the study when measured by the three fixed filters (320/20, 420/20 and 460/30), spectrometer and META spectral detector and the calibration curve for the META detector derived from this data (**d**).

Firstly, the LSM510 META was used to calculate the 500 to 550 nm emission intensity vs excitation wavelength for fluorescein (Fig. 4a). This was compared to literature data^22^, and the ratio derived (Fig. 4b). The results indicate a slight fall in the signal detected by the META detector with excitation wavelengths between 860 and 940 nm and again between 960 and 980 nm, which is contrary to the systematic bias required to cause SHG intensity to apparently increase with excitation wavelength if, in fact, it falls.

A second experiment measured the spectral output from a standard LED torch using a NIST-calibrated Ocean Optics USB2000 spectrometer, the three fixed filters and the META detector. The spectrum determined by the two systems is very similar (Fig. 4c), with the exception of short wavelengths where the LEDs lack output power and so detector dark level dominates. The fixed filters also show a similar response. Above 430 nm the ratio of META response to spectrometer response is an approximate straight line centred on unity (Fig. 4d). There is no evidence of a systematic over-reading of signal levels by the META relative to the spectrometer, which would be needed to explain an increase in SHG with respect to wavelength as a measurement artefact.

### Modelling wavelength dependency of SHG

**Figure 5:**
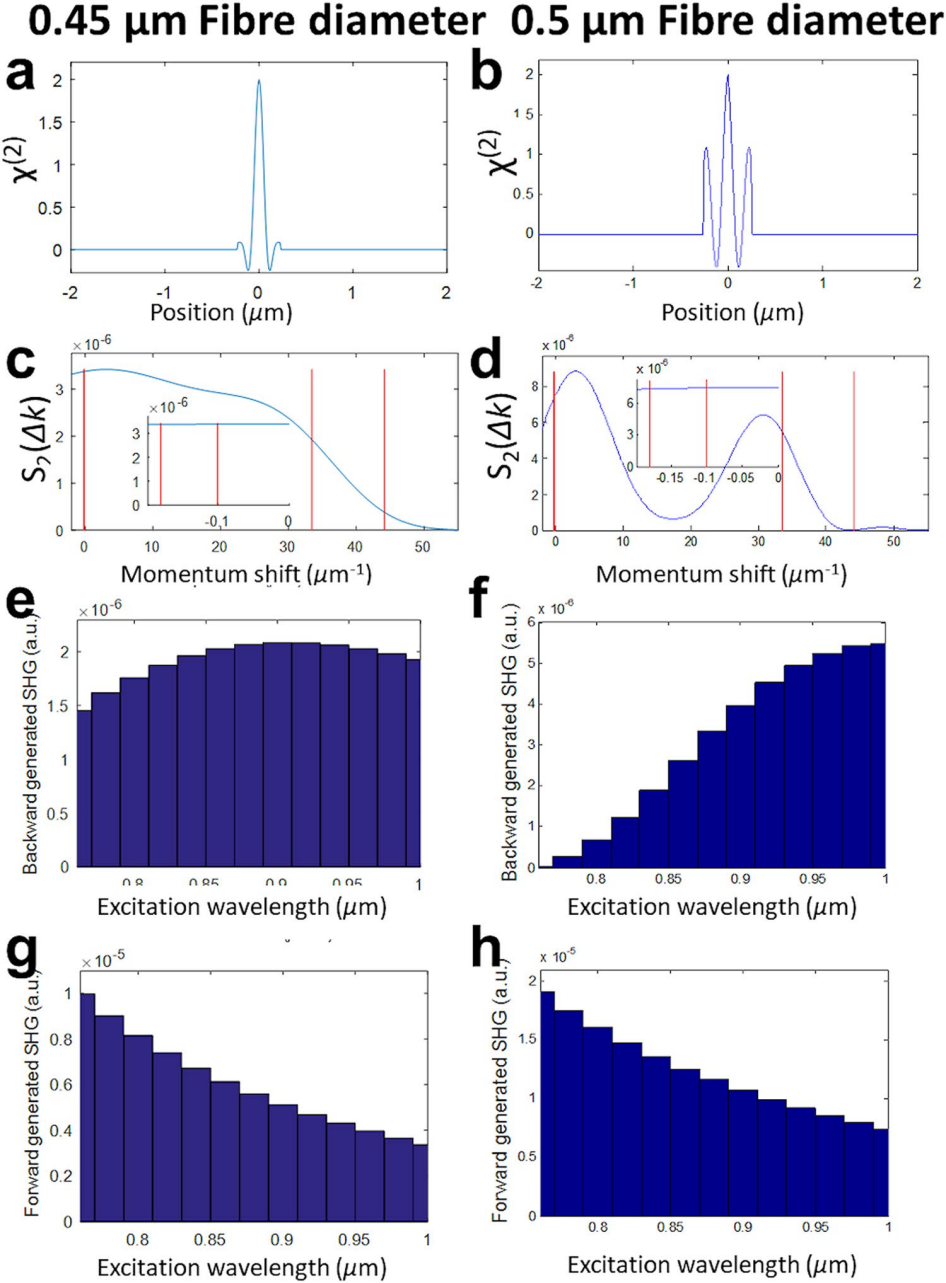
Modelling impact of fibre diameter upon SHG emission. Hypothetical non-random χ^(2)^(*z*) distribution and the resulting non-linear structure factor and predicted forward- and epi-generated excitation spectra for fibres of 0.45 µm (left column) and 0.5 µm (right column) diameter using a 0.23 µm period in χ^(2)^ polarity and the dispersion relation for water. (**a**,**b**) Hypothetical χ^(2)^(*z*) used to generate structure factors. S_2_(Δk) in momentum-space (**c**,**d**). The red vertical lines demarcate the momentum-space corresponding to the spectral range 0.76 to 1.0 micron in the forward- (left) and epi- (right) directions. The inset plot magnifies the range for the forward direction. S_2_(λ)/λ^4^ plotted for the epi- (**e**,**f**) and forward (**g**,**h**) directions respectively.

Figure 5 shows calculations of the predicted SHG intensity $|G{({\boldsymbol{\Delta }}k)}^{2}|/{\lambda }^{4}$ for forward- and epi-generated SHG and two hypothetical distributions χ^(2)^(*z*), representing fibres with 0.45 µm and 0.5 µm diameters and 0.23 µm period in χ^(2)^ in the up/down polarity of the harmonophores. The models show that although this small change in the assumed fibrillar structure of χ^(2)^(*z*) impacts upon the absolute intensity at a given wavelength, it has very little effect on forward-generated SHG wavelength-dependency. For both 0.5 µm and 0.45 µm diameter fibres, there is a monotonic fall in forward-generated SHG intensity with increasing wavelength (Fig. 5g,h). Furthermore, the forward-generated 920 nm/860 nm ratios derived from these models (0.77 and 0.79 for 0.45 and 0.5 µm diameters, respectively) are both very close to 0.76, which would be expected based on a 1/λ^4^ dependency. However, for epi-generated SHG a different response is predicted, with little change in signal intensity across the excitation wavelengths for a 0.45 µm fibre (Fig. 5e), while the predicted epi-generated SHG wavelength-dependency with 0.5 µm fibrillary structure (Fig. 5f) qualitatively reproduces our experimental collagen data (Fig. 2), with an increase in excitation efficiency with increasing wavelength. In addition, the models propose that increasing the fibre diameter from 0.45 µm to 0.5 µm results in a 920 nm/860 nm ratio increase from 1.03 to 1.73. This is qualitatively similar to the measurements using fixed-wavelength filters: the 920 nm/860 nm ratio image for collagen shows an overall dominance of epi-generated SHG intensity at 920 nm, whereas with myosin the two images are of almost identical brightness.

## Discussion

There will obviously be wavelength-dependencies in the optical throughput and temporal pulse-dispersion of the system, which will clearly affect the intensity of a non-linear process^9^. However, external calibration of the system suggests that the data is not significantly affected by variations in response across the wavelength range. Changes in SHG intensity due to collagen type, alignment or amount can also be discounted since the wavelength dependency was observed within the same sample. We also discount wavelength-dependent changes in incident laser polarization relative to a preferred axis in the sample, as there are no discernible differences for fibres with differing orientations. There will be a significant decrease in objective transmission at wavelengths below 400 nm, however this would only affect excitation below 800 nm and cannot account for the majority of the results that are reported in this study.

We hypothesize that the material-dependent differences in SHG wavelength-dependency are due to quasi-phase-matching^23^, which, as our models show, can produce a greater wavelength variation in epi- than forward-generated SHG. Our models illustrate that these differences in epi-generated SHG wavelength-dependency are impacted by small differences in the fibrillar structure of the material under investigation. However, with forward-generated SHG our models indicate that, although the same structural changes impact upon absolute intensity at a given wavelength, they have very little effect on wavelength-dependency beyond the expected monotonic fall.

It is however very important to note that the models presented here are intended primarily to illustrate the potential impact of such small changes on SHG wavelength-dependency in both epi- and forward directions; they are in no way intended to be predictive of the actual spatial structure of the collagen and myosin fibres in the samples. Further research is required to optimize the model parameters to facilitate fully accurate predictions. Potentially this would involve a combination of angle, polarization and wavelength-resolved measurements. However, collagen fibres do associate into bundles and so, in general, have a greater diameter than myosin fibres (e.g. 100–500 µm^24^ for type I collagen in tendon, compared to 3–70 µm^25^ for myosin in muscle).

We have demonstrated experimentally that SHG can carry spectroscopic information sufficient to discriminate different biological samples. Thus, by obtaining ratiometric SHG images at 920 nm and 860 nm, additional information on the nature of the SHG-generating components can be obtained. Furthermore, previous reports of strong wavelength-dependent SHG efficiency should not be discounted as system artefacts because we have shown that a theoretical rationale exists to explain such differences, particularly with epi-generated SHG, even when χ^(2)^ is independent of wavelength. Conflicting accounts of wavelength dependency for epi-detected SHG may also be a result of variations in the proportions of epi-generated and backward-reflected forward-generated SHG within the signal detected, since, as we have shown, backward-reflected forward-generated SHG will have minimal wavelength dependency.

Our work shows the potential of this new source of contrast in SHIM; myosin within smooth muscle tissue and collagen from the surrounding connective tissue can be identified separately. Further work however is required to determine if these results can be reproduced with a range of collagen samples, including non-fibrous structures such as collagen gels. The technique might also be extended to allow SHIM to identify other SHG-generating biomolecules such as microtubules. Additionally, we hypothesize that with optimization of the model parameters, ratiometric information from SHIM at different wavelengths could provide additional structural information, in a similar manner to the ratio of forward- to epi-generated signal^26^.

## Methods

All materials were purchased from Sigma Aldrich (Dorset, UK) unless otherwise stated.

### Human Dermal Fibroblast (HDF) Culture

Normal HDFs were isolated from split thickness skin as described previously^27^, and cultured in fibroblast culture medium. (DMEM, 10% foetal calf serum, 2 × 10^−3^ M glutamine, 100 IU/mL penicillin, 100 µg/mL streptomycin and 0.625 µg/mL amphotericin B). HDFs were used between passages 4 and 11.

HDF were seeded at 1 × 10^5^ cells per well in a 6-well plate, in fibroblast culture medium supplemented with ascorbic acid-2-phosphate (50 µg/ml) and incubated for 21 days, replacing the supplemented medium every 2 or 3 days.

### Ethical approval

Human tissue samples were obtained with informed consent and appropriate ethical approval (Human Tissue Bank licence no. 12179), anonymised and used in accordance with this licence.

### Second Harmonic Generation Imaging using Spectral Detector

SHG images were obtained using an EC Plan Neofluar 40x oil objective (NA 1.3) on a Zeiss LSM510 META upright confocal microscope, equipped with a tuneable femtosecond pulsed Chameleon Ti:sapphire two-photon laser. SHG signal was collected in the backwards scattering, epi-direction (our system does not provide wavelength-resolved measurement in the forward-direction) using the HFT KP650 primary dichroic filter and META detector. Samples of bovine tendon, at least 1 cm thick, were imaged at depths of 10–40 µm using wavelengths between 760 and 1000 nm, in 20 nm increments. The laser power at the sample, measured by a thermal power meter below the objective, was kept constant throughout (10 mW). The pinhole was set to maximum and conditions were kept constant in terms of pixel number (512 × 512), amplifier gain (1) and offset (0.01), detector gain (604), pixel dwell time (6.39 µs) and averaging (x2). For each sample a spectral stack was generated from the signal between 362 and 704 nm, in 32 channels with 10.8 nm resolution.

### Second Harmonic Generation Imaging using Fixed Wavelength Filters

SHG images were obtained as described above except the SHG signal was collected, again in the epi- direction, using the commercial, turn-key LSM510 PMT detector and one of the following narrow band filters; ET390/20x, ET 420/20x or ET460/30x (Chroma Technology Corp, VT, USA). All other settings were kept constant for each sample. The samples imaged were type I collagen generated by human dermal fibroblasts over 21 days in culture, bovine tendon, and myosin from chicken breast muscle. Combined samples of myosin and tendon were imaged using differing laser powers for the two regions (60 mW and 10 mW respectively), due to variations in the intensity of the SHG signal from the two components.

### Image Processing and Analysis

Image analysis used ImageJ^20^ to determine the mean fluorescent intensity for the whole field of view for each channel. The channels containing the SHG signal produced by 860 nm and 920 nm excitation were isolated and combined post-imaging. After image registration, ratio images were displayed by producing a composite RGB image. The 920-nm image was assigned to the red and blue channels, and the 860-nm image to the green channel. Thus, pixels producing a greater signal from 920 nm excitation appear purple and pixels with a greater signal from 860 nm appear green. Where there is no difference in intensity the pixels appear greyscale.

### Analytical and numerical details of the modelling

By making the slowly-varying amplitude approximation (namely that the amplitude of the second harmonic wave – but not necessarily the hyperpolarizability – varies only slowly over the scale of one wavelength), Boyd describes the electric field associated with the second harmonic signal generated by a focused Gaussian beam as: -

4 $${A}_{2}(r,z)=\frac{{A}_{2}(z)}{1+i\zeta }\exp (-\frac{2{r}^{2}}{{w}_{0}^{2}(1+i\zeta )})$$

${w}_{0}^{2}=\lambda /\pi NA$ is the Gaussian beam waist. The axial variation of the amplitude, ${A}_{2}(z)$, obeys the ordinary differential equation: -

5 $$\frac{d{A}_{2}}{dz}=\frac{i\omega }{{n}_{2}c}{\chi }^{(2)}{U}_{0}^{2}\frac{\exp (i\Delta kz)}{1+i\zeta }$$

*U*_0_ defines the electric field amplitude at the Gaussian focus. For an infinite inhomogeneous medium, this equation can be integrated directly to yield:

6 $${A}_{2}=\frac{i\omega }{{n}_{2}c}{U}_{0}^{2}{\int }_{-\infty }^{+\infty }{\chi }^{(2)}(z)\frac{\exp (i\Delta kz)}{1+i\zeta }dz=\frac{i\omega }{{n}_{2}c}{U}_{0}^{2}G(\Delta k)$$

which serves to define the 1-D non-linear structure factor *G*(Δk). It can be shown that the cone angle of the SHG emission equals  $1/\sqrt{2}$ that of the excitation cone angle and hence, if the excitation and detection NA’s are equal (as is the case for epi-detection), then the total detected SHG equals the intensity integrated across the beam cross-section at the Gaussian focus i.e.

7 $${I}_{SHG}\propto \frac{{\omega }^{2}{U}_{0}^{4}{|G({\rm{\Delta }}k)|}^{2}}{1+{\zeta }^{2}}{{\int }_{0}^{\infty }|\exp (-\frac{2{r}^{2}}{{\omega }_{0}^{2}(1+i\zeta )})|}^{2}2\pi rdr\propto \frac{{|G(\Delta k)|}^{2}}{{\lambda }^{4}}$$

Figure 5c,d were calculated for two hypothetical χ^(2)^(*z*) distributions (5a and 5b). A unit-amplitude cosinusoidal oscillation of period 0.23 microns is apodized by a Hamming window of diameter 0.45 microns (5a) or a top-hat function of width 0.5 microns (5b). In both cases a unit-amplitude Hamming function is then added, to provide a consistent and numerically stable phase-matching peak near the origin.

To maintain an adequate momentum-space sampling rate, especially for forward-generated SHG, we zero-pad the spatial window out to 96 µm and discretize into N = 8192 pixels. Hence the spatial sampling rate of 85.33 µm^−1^ corresponds to a maximum unaliased Δk of 268 µm^−1^, with a momentum-space sampling interval of 0.065 µm^−1^. The maximum range is more than adequate to accommodate the quasi-phase-matching peak at 44 µm^−1^ when calculating the epi-generated spectrum, whilst the sampling rate provides four distinct calculated values of the forward-generated spectrum across our excitation wavelength range, with the full spectrum then being evaluated by interpolating between these values.

### Data availability

The datasets generated and/or analysed during the current study are available from the University of Sheffield online data repository https://orda.shef.ac.uk/.
